# Minimal-Activity 18F-FDG PET/CT as a Potential Problem-Solving Tool in a Patient with Suspected Lung Cancer Recurrence

**DOI:** 10.3390/diagnostics16121789

**Published:** 2026-06-10

**Authors:** Theresa Leder, Nathalie Viohl, Christian Kühnel, Falk Gühne, Martin Freesmeyer

**Affiliations:** Clinic of Nuclear Medicine, Jena University Hospital, 07747 Jena, Germany

**Keywords:** minimal activity, segmental PET/CT, non-small cell lung cancer, 18F-FDG, radiation exposure, cancer staging

## Abstract

A 74-year-old woman with a history of non-small cell lung cancer underwent 18F-FDG PET/CT to rule out recurrence of the disease. The whole-body scan revealed a nodule in the left upper lobe of the lung that could not be unequivocally distinguished as either malignant or inflammatory. A segmental minimal activity (MA) PET/CT was performed for clarification, as it offers additional metabolic information without significantly increasing radiation exposure. Based on the MA-PET/CT scan and clinical findings, the nodule was classified as inflammatory. To the best of our knowledge, the use of MA-PET/CT in cases of suspected lung cancer recurrence has rarely been reported so far.

**Figure 1:**
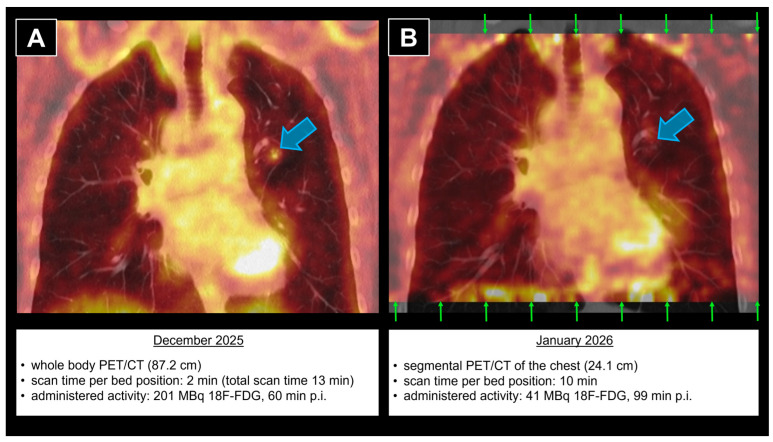
A 74-year-old woman with non-small cell lung cancer (NSCLC) presented to our clinic to exclude disease recurrence. NSCLC of the left upper lobe was first diagnosed in March 2019. Segmental resection of the tumor had been performed with curative intent. Histology revealed stage IA2 adenocarcinoma (pT1b pN0 cM0) according to IASLC criteria [1,2]. So far, follow-up examinations had been without pathological findings. An 18F-FDG PET/CT scan was conducted to exclude cancer recurrence in the context of non-specific pulmonary symptoms. The administered activity was 201 MBq 18F-FDG. The total scan range was 87.2 cm, with a total scan duration of 13 min (two minutes per bed position). The scan demonstrated a new, slightly hypermetabolic nodule in the remaining left upper lobe of the lung (**A**: coronal PET/CT scan of the chest; blue arrow indicates the nodule with low tracer uptake (SUVmax 2.4) and a maximum diameter of 5 mm). No additional suspicious findings were detected. Reliable distinction of the pulmonary lesion as either cancer recurrence or inflammatory consolidation was not possible, particularly since the patient’s clinical symptoms were also consistent with a respiratory tract infection. Further diagnostic clarification was required. Transbronchial biopsy was deemed inappropriate due to the small lesion size, which rendered it susceptible to sampling error [3,4]. Instead, another PET/CT scan was scheduled on the patient’s explicit request to help confirm the diagnosis. In accordance with the ALARA principle, efforts were made to minimize the patient’s radiation exposure during the follow-up examination. Therefore, given the isolated finding, the second examination was performed as a segmental minimal-activity (MA) PET/CT. MA-PET/CT constitutes a technical modification, limiting the field of view to one bed position: The patient’s radiation exposure is reduced by restricting the CT scan area and decreasing the administered tracer activity. The lower count statistics can be compensated for by extending the scan duration. The technique was first trialed on models or scenarios and has since been adopted in clinical practice [5,6]. After eight weeks and subsequent to the patient’s full recovery from potentially pneumonic symptoms, MA-PET/CT of the chest was performed according to an internal clinical protocol (**B**: coronal MA-PET/CT scan of the chest; blue arrow indicates the pulmonary nodule with decreasing hypermetabolism (SUVmax 1.0), size and density; green arrows indicate the borders of the limited scan area). The administered activity was 41 MBq 18F-FDG. The scan range was 24.1 cm and the scan duration was 10 min. The MA-PET/CT examination resulted in a patient’s total effective radiation dose of 1.87 millisieverts (mSv). The examination revealed metabolic and morphological regression of the pulmonary lesion. Nevertheless, the results must be interpreted with caution, as partial volume effects (PVEs) can essentially influence the metabolic and morphological impression, especially in small lesions. Visual and quantitative (i.e., SUVmax) analysis is therefore limited [7]. So far, PVE cannot be eliminated entirely, although standardized acquisition modalities (spatial resolution, voxel size) contribute to effectively reducing its impact. Nevertheless, PVE is a shortcoming that may be overcome with the help of deep learning tools in the future. Deep learning-based reconstruction can contribute to more accurate acquisition and better image quality, consequently enhancing diagnostic accuracy. Recently, frameworks have been developed to predict partial-volume-corrected full-dose images from either standard or low-dose PET images, but they have not yet been adopted in clinical practice [8]. With regard to the clinical course, the pulmonary lesion was retrospectively categorized as an inflammatory consolidation. However, lung cancer recurrence cannot be ruled out completely, due to lacking histology, but seems to be rather unlikely. No further follow-up examinations have been conducted to date. MA-PET/CT can be an effective, cost-efficient tool to determine the origin of localized findings [9,10]. It provides metabolic information equivalent to a whole-body PET/CT, enabling a second evaluation of inconclusive results, while comparable to the radiation dose of a low-dose chest CT [10,11]. Moreover, the restricted scan range offers additional protection for radiosensitive organs compared to whole-body PET/CT [6]. This case study demonstrates the potential use of MA-PET/CT in evaluating ambiguous findings in suspected lung cancer recurrence. However, given the nature of this single case report, no general conclusions can be drawn from these findings. To the best of our knowledge, few reports have addressed the use of MA-PET/CT in this specific clinical setting so far. Advances in imaging technology as well as implementation of deep learning and standardized examination protocols may further enhance the potential usefulness of this technique. The intention of this report was therefore not to establish definitive clinical implications, but rather to stimulate further discussion and evaluation of MA-PET/CT in patients with suspected lung cancer recurrence.

## Data Availability

No new data were created or analyzed in this study. Data sharing is not applicable to this article.

## Abbreviations

The following abbreviations are used in this manuscript:

NSCLC	Non-small cell lung cancer
IASLC	International Association for the Study of Lung Cancer
18F-FDG	2-Fluor-2-desoxy-D-glucose
PET	Positron emission tomography
CT	Computed tomography
cm	Centimeter
min	Minute
SUVmax	Maximum standardized uptake value
ALARA	As low as reasonably achievable
MA	Minimal activity
mSv	Millisieverts
PVE	Partial volume effect
